# Effects of Natural Flavonoids on Photosynthetic Activity and Cell Integrity in *Microcystis aeruginosa*

**DOI:** 10.3390/toxins7010066

**Published:** 2015-01-09

**Authors:** Haomin Huang, Xi Xiao, Anas Ghadouani, Jiaping Wu, Zeyu Nie, Cheng Peng, Xinhua Xu, Jiyan Shi

**Affiliations:** 1 College of Environmental & Resource Science (CERS), Zhejiang University, Hangzhou 310058, Zhejiang, China; E-Mails: skeeterhuang@zju.edu.cn (H.H.); niezeyu@zju.edu.cn (Z.N.); zjupcsnow@gmail.com (C.P.); xuxinhua@zju.edu.cn (X.X.); 2 Ocean College, Zhejiang University, Hangzhou 310058, Zhejiang, China; E-Mail: jw67@zju.edu.cn; 3 Aquatic Ecology and Ecosystem Studies, M015, School of Civil, Environmental Systems and Mining Engineering, the University of Western Australia, 35 Stirling Highway, Crawley, WA 6009, Australia; E-Mail: anas.ghadouani@uwa.edu.au

**Keywords:** flavonoid, cyanobacteria, pulse-amplitude modulation, flow cytometry, photosynthesis

## Abstract

Flavonoids are natural polyphenolic compounds produced by many aquatic plants and released in their environments. In this study, the effects of several aquatic flavonoids on cyanobacterial *Microcystis aeruginosa*, especially in relation to the cell growth, photosynthetic activity, cell morphology, and cell membrane integrity, were investigated. Significant growth inhibition was observed when the cyanobacteria were exposed to three flavonoids, namely, 5,4'-dihydroxyflavone (DHF), apigenin, and luteolin. Luteolin reduced the effective quantum yield, photosynthetic efficiency, and maximal electron transport rate by 70%, 59% and 44%, respectively, whereas 5,4'-DHF and apigenin slightly affected these parameters, which implies that luteolin disrupts the photosynthetic system. Moreover, 5,4'-DHF and apigenin compromised the membrane integrity, and induced membrane depolarization in 52% and 38%, and permeabilization in 30% and 44% of the cells, respectively. The 5,4'-DHF and apigenin showed more pronounced effects on *M. aeruginosa* morphology and membrane integrity, compared to the luteolin. These results suggest that flavonoids could have significant effects on growth and physiological functions in cyanobacterial species.

## 1. Introduction

Flavonoids are low molecular weight natural polyphenolic chemicals that play important roles in various metabolic processes in photosynthesizing cells, and their existence is therefore widespread in the plant kingdom [1]. Chemically, flavonoids have the basic structure of a chromone (1,4-benzopyrone) moiety connected to a phenyl ring at position 2. Over 5000 flavonoids, from different tissues and parts of plants, have been characterized, including leaves, flowers, stems, pollen and seeds [2]. While multiple biological effects of flavonoids derived from terrestrial plants have been studied since they were identified as “vitamin P” in the mid-1930s [3,4], flavonoids from aquatic plants have only been investigated in the recent decade. Various flavonoids are produced by common aquatic plants, *i.e.*, *Posidonia oceanica* [5], *Nelumbo nucifera* [6], *Azolla microphylla* [7], and *Eichharnia crassipes* [8]. Usually the content of flavonoids in aquatic plants ranged from 0.005% to 1.2% of the plant dry mass [6,9,10,11]. Flavonoids are released into the environment by growing plants especially when they are under physiological stress, or from decomposing parts of plants. Flavonoids have multiple unique biological properties, including antioxidant, anti-inflammatory, antibacterial, antifungal effects just to name a few [12,13]. Many studies focused on the effects of flavonoids on microbes in medicine or the food industry, however; their interactions with aquatic organisms in the natural environment are not adequately studied.

As the most ancient photosynthetic species, cyanobacteria have existed in aquatic environments for ~3500 million years. The high nutrient utilization efficiency of cyanobacteria acquired over their long evolutionary history is partly responsible for their ability to adapt to extreme aquatic environments, ranging from polar to tropic regions, and from freshwater to marine environments. Allelopathy, a mechanism by which plant could influence other organisms, including other plants, through the production and release of allelochemicals, has been suggested as way plants can interact with cyanobacterial species [14]. However, the effects of flavonoids, as a common allelochemicas from higher plants, on cyanobacteria, are yet to be understood.

On one hand, cyanobacteria may accumulate flavonoids under stress to protect cellular damage [15]. On the other hand, a few studies suggest that extracts of plants containing flavonoids could have negative effects on the growth of cyanobacteria [16,17]. For example, an aqueous extract of the *Ephedra equisetina* root was found to induce cyanobacterial cell death, presumably because it contained a high concentration of flavonoid compounds [18,19]. Only one study reported that flavonoids inhibit the growth of harmful algae, in which Li *et al.* [20] found that baicalein and baicalin, active ingredients of *Radix Scutellariae* (dried root of the medicinal plant *Scutellariae baicalensis*), significantly suppressed *Karenia mikimotoi* and *Chattonella marina* growth with IC_50_ values of 12.6 and 32.6 mg/L. In a previous study, we also found that the flavonolignans salcolin A and salcolin B in barley straw inhibited cyanobacteria growth and induced an increase on intracellular ROS levels and esterase activity suppression [21,22]. The specific biological responses of cyanobacterial cell systems (*i.e.*, photosynthesis system or membrane system), in the flavonoid-cyanobacteria interactions are yet to be elucidated. As flavonoids are of the category of polyphenolic which effects on cyanobacteria have been only partly investigated; flavonoids, like other polyphenols, may interfere with the photosynthetic chain, disrupt plasma membrane integrity, and lead to oxidative damage [23,24,25].

In this study, we investigate the biological responses of cell systems, especially the photosynthesis and membrane system, in an environment where flavonoids and cyanobacteria coexisted. We performed a series of batch experiments in microcosms containing the cyanobacteria *M. aeruginosa* and three natural flavonoids, namely 5,4'-dihydroxyflavone (DHF) from *Lancea tibetica*, apigenin and luteolin from *Elodea* species, which are the flavonoid derivatives of salcolins extracted from barley straw (*Hordeum vulgare*) and used in our previous study [22]. The effects of the addition of flavonoids was assessed through the measurement of (i) the changes in the growth of *M. aeruginosa*; (ii) changes in the cyanobacterium’s photosynthetic activity; as well as (iii) the impact on cell integrity.

**Figure 1:**
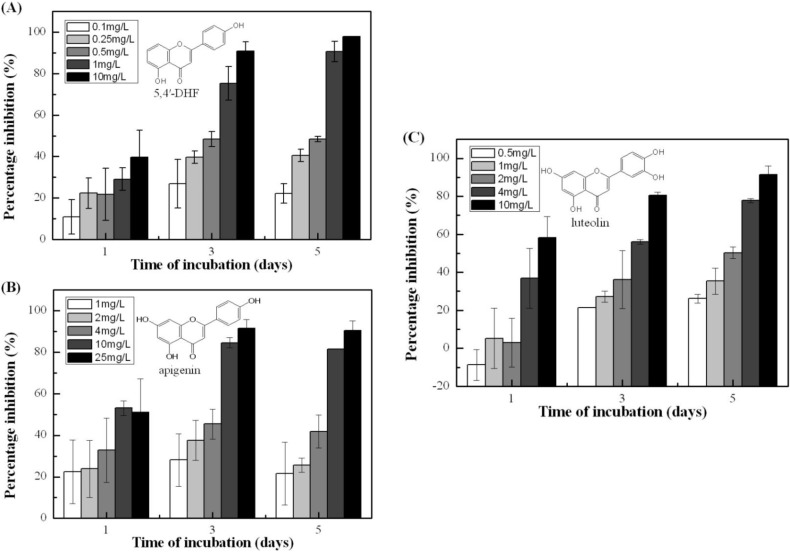
Growth inhibition of *Microcystis aeruginosa* by different flavonoids. (**A**): Effect of 5,4'-DHF with concentrations of 0.1, 0.25, 0.5, 1, and 10 mg/L; (**B**): Effect of apigenin with concentrations of 1, 2, 4, 10, and 25 mg/L; (**C**): Effect of luteolin with concentrations of 0.5, 1, 2, 4, and 10 mg/L. Error bars indicated one standard deviations from the mean based on three replicates.

## 2. Results

### 2.1. Growth Inhibition Effects of 5,4'-dhf, Apigenin and Luteolin

Microscopic analyses revealed that the three flavonoids showed significant concentration-dependent inhibition of *M. aeruginosa* growth (*p* < 0.05) after 5-d incubation (Figure 1). After one day of exposure, all three flavonoids slightly inhibited *M. aeruginosa* growth under low concentrations. Three-day exposure produced stronger inhibition activity. After 5 days, the cyanobacterial biomass in the treated groups declined to the lowest density for the maximum concentration (10 mg/L 5,4'-DHF, 25 mg/L apigenin and 10 mg/L luteolin based on the preliminary experiments), and the inhibition rates were 97.90% ± 0.01%, 90.37% ± 4.65%, and 91.54% ± 4.62%, respectively. 5,4'-DHF, apigenin, and luteolin exhibited high anti-cyanobacterial activity but differed in their activities. The EC_50_ values of 5,4'-dihydroxyflavone (DHF), apigenin, and luteolin were 0.47, 3.85, and 1.85 mg/L for five days, respectively (see Table S1). Therefore, different concentrations were used for the individual flavonoids according to their EC_50_ values, *i.e.*, the treated concentrations of 5,4'-DHF were 0.25, 0.5, and 1 mg/L; those of apigenin were 2, 4, and 8 mg/L; and those of luteolin were 1, 2, and 4 mg/L.

**Figure 2:**
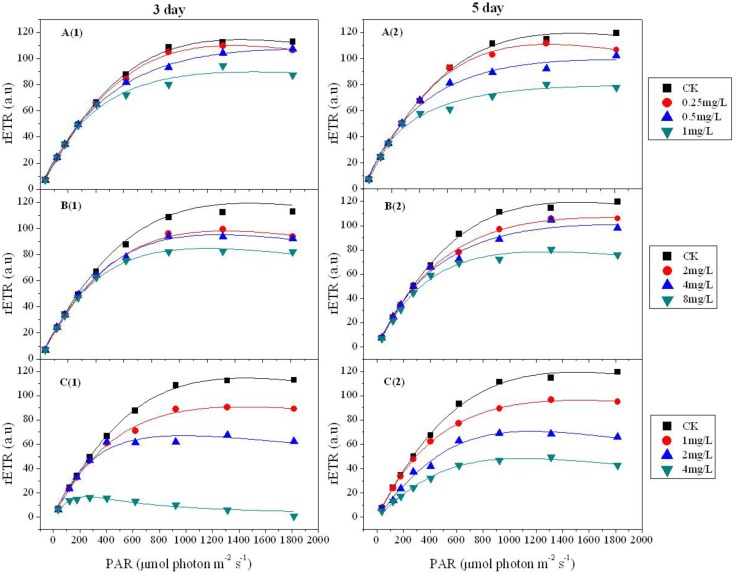
Photosynthesis *versus* irradiance (rETR/E) curves for *Microcystis aeruginosa* treated with 5,4'-DHF (**A**); apigenin (**B**); and luteolin (**C**). Each observation represents the mean of three different experiments and the solid line represents model results. Comparisons were performed using the method of Ratkowski [26] for non-linear models as explained in the experimental section.

### 2.2. Inhibition of Cyanobacterial Photosynthetic Activity by Flavonoids

The photosynthetic efficiency of flavonoid-treated cyanobacteria was further examined using PAM fluorometry and the rETR/E (RCL) curves of the three flavonoids were compared. Figure 2 shows that the three flavonoids, especially luteolin, significantly decreased (*p* < 0.05) the rETR in response to PAR (actinic photosynthetically active radiation generated by PAM), indicating negative effects on the photosynthesis system of *M. aeruginosa*.

**Figure 3:**
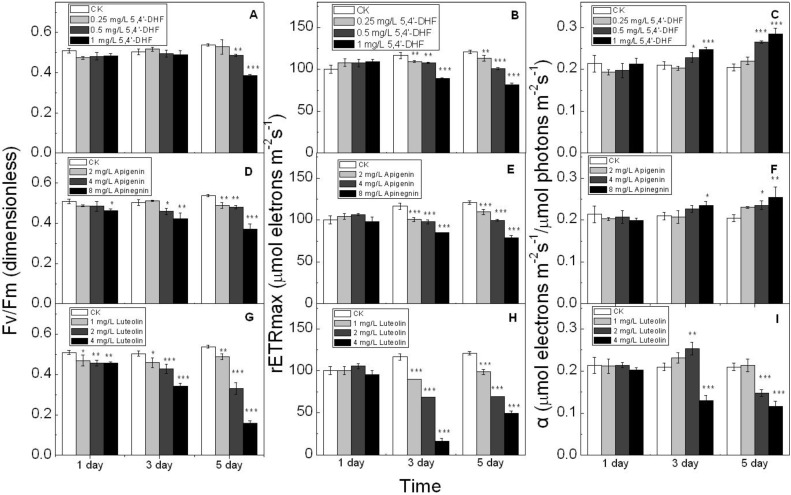
Photosynthetic parameters of *Microcystis aeruginosa* exposed to 5,4'-DHF (**A**–**C**); apigenin (**D**–**F**); and luteolin (**G**–**I**) for 1, 3, and 5 days (left: *F_v_*/*F_m_*, effective quantum yield, dimensionless; middle: rETR_max_, maximal electron transport rate, μmol electrons m^−1^s^−2^; right: α, photosynthetic efficiency). *, ** and *** represent statistically significant differences with *p* < 0.05, *p* < 0.01 and *p* < 0.001 relative to the control without flavonoids. Error bars indicate one standard deviation from the mean.

The changes in the effective quantum yield *F_v_*/*F_m_* and other photosynthetic parameters over time calculated from the RCLs, including the photosynthetic efficiency alpha and maximal electron transport rate rETR_max_, are shown in Figure 3. Exposure to 0.25 mg/L 5,4'-DHF for 5 d did not elicit significant changes (*p* > 0.05). However, increasing the 5,4'-DHF concentration to 0.5 and 1 mg/L significantly affected all three photosynthetic parameters (*p* < 0.05) (Figure 3A–C). After 5 d of 1 mg/L 5,4'-DHF exposure, *F_v_*/*F_m_* and rETR_max_ were significantly (*p* < 0.01) decreased from 0.54 (control) to 0.38 and from 121.0 (control) to 81.3 μmol electrons m^−2^ s^−1^, respectively, whereas the photosynthetic parameter alpha, *i.e.*, the photosynthetic rate in the light-limited region, considerably increased (from 0.20 to 0.28 μmol electrons m^−2^ s^−1^/μmol photons m^−2^ s^−1^; *p* < 0.01).

The effect of apigenin on the three photosynthetic parameters appeared to be similar to that of 5,4'-DHF (Figure 3D–F). Increase of the apigenin concentration to 4 mg/L caused decreases from 0.5 (control) to 0.46 (*p* < 0.05) in *F_v_*/*F_m_* and from 116.7 (control) to 97.9 μmol electrons m^−2^ s^−1^ (*p* < 0.01) in rETR_max_, whereas alpha increased from 0.21 to 0.23 μmol electrons m^−2^ s^−1^/μmol photons m^−2^ s ^−1^ (*p* < 0.05) within 3 d of exposure. After another 2 d, *F_v_*/*F_m_* and rETR_max_ further decreased to 0.42 (*p* < 0.05) and 89.8 μmol m^−2^ s^−1^ (*p* < 0.01), respectively, whereas alpha increased to 0.24 μmol electrons m^−2^ s^−1^/μmol photons m^−2^ s^−1^ (*p* < 0.05). Similarly, *F_v_*/*F_m_* and rETR_max_ declined and alpha increased after treatment with 8 mg/L apigenin.

The influence of luteolin on the photosynthesis in *M. aeruginosa* was shown to be more conspicuous than that of the other flavonoids (Figure 3G–I). Specifically, 4 mg L^−1^ luteolin reduced *F_v_*/*F_m_* from 0.50 (control) to 0.34 (*p* < 0.01) after 3 d, and from 0.54 (control) to 0.16 (*p* < 0.01) after 5 d. The inhibition of rETR_max_ was even more significant. After 3 and 5 d of treatment, 4 mg L^−1^ luteolin reduced rETR_max_ from 121.0 (control) to 49.6 (*p* < 0.01) μmol electrons m^−2^ s^−1^, and from 116.7 (control) to 16.3 (*p* < 0.01) μmol electrons m^−2^ s^−1^. The effects of luteolin on alpha were different from those of the other flavonoids. Compared to the values of 0.21 (3 d) and 0.20 (5 d) μmol electrons m^−2^ s^−1^/μmol photons m^−2^ s^−1^ in the control groups, 2 mg L^−1^ luteolin first increased alpha to 0.23 (*p* < 0.05; 3 d) μmol electrons m^−2^ s^−1^/μmol photons m^−2^ s^−1^ and then decreased it to 0.15 (*p* < 0.01; 5 d) μmol electrons m^−2^ s^−1^/μmol photons m^−2^ s^−1^, whereas at 4 mg L^−1^ luteolin continuously decreased alpha to 0.13 (3-d) and 0.12 (5-d) μmol electrons m^−2^ s^−1^/μmol photons m^−2^ s^−1^.

### 2.3. Effects of Flavonoids on the Cell Morphology and Membrane Integrity of M. aeruginosa

**Figure 4:**
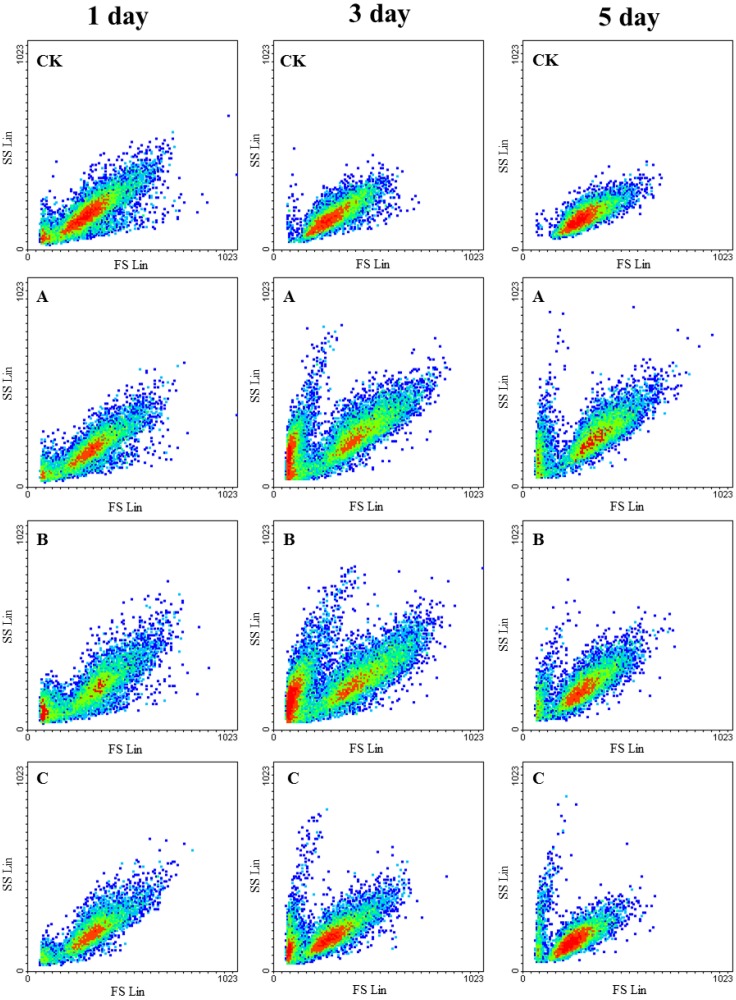
Cytograms of *Microcystis aeruginosa* exposed to various flavonoids (**A**: 5,4'-DHF; **B**: apigenin; **C**: luteolin) or not (CK: control) depending on FSC and SSC for 1, 3, and 5 days.

The morphological characteristics of *M. aeruginosa* cells were assessed by flow cytometry; FSC (forward scattered light) and SSC (sideward scattered light) signals provided information regarding cell size and granularity, respectively. The changes in the size and internal complexity of *M. aeruginosa* cells treated with 5,4'-DHF, apigenin, and luteolin are shown in Figure 4. After three days exposure to 5,4'-DHF and apigenin, two distinct subpopulations of *M. aeruginosa* were observed: one consisted of shrunken cells and cell debris with high granularity, whereas the other consisted of normal or large swollen granular cells. The cell size in the first subpopulation decreased by 35.1% and 45.5%, whereas the cell size in the second population increased by 31.0%and 32.6% after three days of exposure to 1 mg/L 5,4'-DHF and 8 mg/L kapigenin, respectively (See Table S2). *M. aeruginosa* morphology seemed to partially recover after five days. However, the change in morphology induced by luteolin treatment was slight when compared to those induced by 5,4'-DHF and apigenin.

Analysis of the DiBAC_4_(3)-derived fluorescence in *M. aeruginosa* exposed to increasing concentrations of 5,4'-DHF, apigenin, or luteolin showed that flavonoids caused depolarization of the plasma membrane in most cyanobacteria (Table 1). After short-term exposure of one day to 2 and 4 mg/L apigenin, more than 30% of cyanobacterial cells exhibited membrane depolarization. After three days of flavonoid treatments, the proportion of depolarized cells significantly increased, especially in the 1 mg/L 5,4'-DHF treated group, with 52.25% of cells being depolarized. However, a longer exposure of five days diminished the degree of *M. aeruginosa* polarization. These findings demonstrate that the flavonoids caused only short-term changes in the membrane potential of *M. aeruginosa*.

**Table 1:** Comparison of cell membrane depolarization and integrity of *Microcystis aeruginosa* cells after 1-, 3-, and 5-days of exposure.

Flavonoids	Concentration (mg/L)	1 day		3 days		5 days	
		Depolarized ^a^	Permeabilized ^b^	Depolarized	Permeabilized	Depolarized	Permeabilized
Blank Control	0	6.67 (0.11) ^c^	21.70 (1.98)	10.62 (0.37)	16.42 (0.13)	4.47 (1.30)	1.74 (2.41)
5,4''-DHF	0.25	2.49 (0.06)	27.45 (0.21) *	17.26 (0.70) *	20.39 (0.24) *	7.25 (0.43)	5.90 (0.45) *
	0.5	6.66 (0.64)	28.50 (0.14) *	24.06 (1.57) **	28.22 (0.54) *	25.51 (0.53) **	6.29 (0.01) *
	1	6.36 (2.12)	26.30 (0.28) *	52.25 (0.16) ***	30.63 (0.48) **	42.40 (0.05) ***	5.9 (0.04) *
Apigenin	2	2.63 (0.26)	27.80 (0.28) *	12.99 (0.96) *	37.92 (0.08) **	5.46 (0.44)	6.23 (0.04) *
	4	33.54 (3.17) ***	33.85 (0.21) *	15.73 (0.86) *	40.45 (0.73) ***	21.17 (0.44) **	14.57 (0.47) **
	8	37.24 (1.54) ***	37.05 (0.35) *	38.14 (1.40) **	44.42 (0.30) ***	20.85 (0.47) **	17.53 (0.81) **
Luteolin	1	6.71 (0.18)	21.45 (0.78)	23.18 (0.49) **	20.11 (0.44)	6.55 (1.01)	5.70 (0.11) *
	2	7.06 (0.06)	22.25 (0.21)	23.07 (0.50) **	20.50 (2.34)	5.81 (0.89)	9.29 (0.37) *
	4	6.67 (0.16)	22.40 (0.56)	34.18 (1.86) **	25.19 (0.51) *	6.82 (0.44)	9.88 (0.38) **

^a^ Cell membrane depolarization indicated as the percentage of DiBAC_4_(3)-stained cells (with depolarized membrane) obtained by the events occurring in the regions derived from populations of the total amount of cells analyzed by flow cytometry; ^b^ Cell membrane integrity expressed as the percentage of PI-unstained cells (with intact cell membrane) with respect to the total amount of cells analyzed by flow cytometry; ^c^ The values indicate the mean of three tests and one standard deviation in parentheses. A minimum of 5000 cells were analyzed for each test; *, ** and *** represent statistically significant differences of *p* < 0.05, *p* < 0.01 and *p* < 0.001 when compared to the control without flavonoid.

Flow cytometric analysis of PI-stained cells showed that the flavonoids affected the permeability and integrity of the *M. aeruginosa* membrane, as indicated by a significant intracellular PI influx (Table 1). At day 3, 5,4'-DHF and apigenin induced membrane permeability in a significant proportion of *M. aeruginosa* cells with a maximum cell damage rate of 44.42% observed for 8 mg/L apigenin; in contrast, the effect of luteolin was not significant. At day 5, most of the cells had recovered from the flavonoid-induced damage, although 17.53% of cyanobacteria treated with at 8 mg/L apigenin still showed increased membrane permeability.

## 3. Discussion

The results revealed that flavonoids seem to have affected important growth and metabolic characteristics in *M. aeruginosa*, including cell integrity, membrane potential, and photosynthetic capacity. Increasing the flavonoid concentration from 1 to 10 mg/L increased the inhibitory effects on cell growth (Figure 1). Our data are consistent with previous results demonstrating *M. aeruginosa* growth suppression by flavonoids or flavonoid-containing plant extracts [16,17].

For plants and algae, the electron transport rate (rETR_max_) and effective quantum yield (*F_v_*/*F_m_*) in photosystem II (PS II) are restricted by the capacity of the electron transport chain or Calvin activity in photosynthesis [27]. Our finding that luteolin treatment caused a significant reduction in the *F_v_*/*F_m_* and rETR_max_ of cyanobacteria may explain some previously observed interactions of secondary metabolic chemicals from plants with cyanobacteria. In particular, crude extracts of *Zostera marina* that affected negatively the growth of the toxic dinoflagellate *Alexandrium catenella* were later shown to contain the luteolin derivative 7,3'-disulfate luteolin [17,28]. Similarly, luteolin treatment was found to concentration-dependently inhibit *Alexandrium tamarense* and *Prorocentrum donghaiense* [29]. An extract of *A. melanoxylon* flowers containing several flavonoids, including catechin, luteolin, rutin, apigenin, and quercetin, decreased the quantum efficiency of open PSII reaction centers (*F_v_*/*F_m_*) and quantum yield (ФPSII) of photosystem II in the plants *Lolium perenne*, *Dactylis glomerata*, *Rumex acetosa,* and *Lactuca sativa* [30]. The underlying flavonoid-cyanobacteria interactions likely involves the interruption of the electron transport in the PS II reaction center through disruption of the function of the secondary electron acceptor (Q_B_) complex and reduction of the effective quantum yield, which results in impairment of photosynthesis [31].

In this study, the changes in alpha, which reflects the maximum light-limited photosynthesis rate and the efficiency of light capture, exhibited a pattern different from that of the other two parameters; at low concentrations of luteolin, alpha increased, whereas at high concentrations, alpha decreased. These data suggest that, in their presence of low luteolin levels, cyanobacteria could increase the photon transfer and the trapping capacity in the photosynthetic system as an adaptive response to stress, whereas high concentrations lead to breakdown of the entire photosystem. These results suggest that luteolin may interact with the Q_B_-protein complex of PS II to interrupt the electron transport, leading to the failure of photosynthesis. In contrast, the photosynthetic parameters *F_v_*/*F_m_* and rETR_max_ slightly decreased following treatment with 5,4'-DHF or apigenin, whereas alpha showed a relative increase over a 5-d cultivation.

Some interaction of chemicals with cyanobacteria alter cell morphology and compromise membrane integrity, causing leakage of cell components [32]. Our results show that 5,4'-DHF and apigenin strongly affect cyanobacterial morphology and cell membrane permeability (Figure 4 and Table 1). The cytometric plots indicate that cells exposed to moderate concentrations of 5,4'-DHF and apigenin (1.0 and 2.0 mg L^−1^) responded with either shrinking or swelling of the cell volume. Similarly, two distinct subpopulations in algal cultures have been previously observed for *M. aeruginosa* exposed to nanaomycin A methyl ester [33]. Larger cells have lower nutrient uptake rates, causing further nutrient limitation and resulting in the observed lack of growth [34]. Furthermore, the cells that could not adapt to the direct interaction caused by 5,4'-DHF and apigenin experienced membrane disruption and cell shrinkage. This phenomenon was further confirmed by the data on membrane integrity and permeability assessed by DiBAC_4_(3) and PI staining. In the case of microorganisms such as cyanobacteria, perturbations of the cell membrane potential have been suggested as mediators of subsequent physiological cellular responses to environmental stress [35]. Our analysis indicates that treatment with 5,4'-DHF and apigenin affects *M. aeruginosa* membrane polarization. The disruption of the cell membrane in cyanobacteria exposed to flavonoids was associated with membrane depolarization (Table 1), which is consistent with previous findings that methicillin-resistant *Staphylococcus aureus* treated with the flavonoid (3-*O*-octanoyl-(+)-catechin) exhibited highly increased levels of PI staining [36]. These results, together with the data on photosynthesis status, suggest that membrane disruption leads to inhibition of photosynthesis and eventually results in the increasing proportion of death of cyanobacteria as treated with increasing concentration of 5,4'-DHF or apigenin.

Flavonoids with different substitutions in the side chains often exhibit distinct biological and even chemical properties. Although there are no direct reports on the effects of flavonoid structure on the flavonoid-cyanobacteria interactions, structure-functional correlations have been observed in the interactions of flavonoid derivatives with virus and bacteria [37,38]. These studies demonstrated that the different interaction mode of the tested flavonoids with cyanobacteria depend on the substitutions of the side chains. Without an OH group at position 7 in the A-ring, 5,4'-DHF exhibited stronger effects on cell growth suppression. A possible explanation is that the OH group at position 7 significantly increases flavonoid antioxidant capacity [39], which would mitigate the oxidative stress produced by oxygen radicals in cyanobacteria. In contrast, other biological activities of flavonoids seem to benefit from the addition of OH groups in the B ring, as indicated by comparative structure-activity studies involving apigenin (a single OH group at position 4) and luteolin (two OH groups at positions 3 and 4) [40]. A previous study found that the catechol moiety in the flavonoid B ring provides binding sites for iron [41], which is one of the most important trace metal for photosynthesis in cyanobacteria.

## 4. Experimental Section

### 4.1. Cyanobacterial Strains, Culture Medium, and Flavonoids

The *M. aeruginosa* FACHB-469 strain was provided by the Freshwater Algae Culture of Hydrobiology (Wuhan, Hubei, China). Cells were grown in batch cultures in 250-mL Erlenmeyer flasks with sterilized BG11 medium [42] under a 12/12-h light/dark cycle with a light intensity of 70 μM photons/(m^2^s) at 25 ± 1 °C, as previously described [21]. The cultures were used when the microorganisms were in the exponential growth phase. The flavonoid 5,4'-DHF (95%, CAS No. 6665-67-4) was purchased from J&K Scientific Company (Beijing, China), and apigenin (98%, CAS No. 520-36-5) and luteolin (98%, CAS No. 491-70-3) were purchased from Chengdu Must Bio-technology Co., Ltd. (Chengdu, China).

### 4.2. An Assay to Study the Interactions between Flavonoids and Cyanobacteria

*M. aeruginosa* test cultures (1.0 × 10^6^ cells mL^−1^) were placed in 150-mL conical flasks and incubated in BG11 medium containing 5,4'-DHF (0.1, 0.25, 0.5, 1, and 10 mg/L), apigenin (1, 2, 4, 10, and 25 mg/L), and luteolin (0.5, 1, 2, 4, and 10 mg/L) at concentrations determined in preliminary tests (See Table S3). The algal density was determined using a hemocytometer with a microscope, and the percentage inhibition (%) is calculated by the formula given below:

(1) $$\text{Percentage}\;\text{inhitition}\;(\%)=\frac{cell\;density_{\left[control\right]}-cell\;density_{\left[flavonoid-add\right]}}{cell\;density_{\left[control\right]}}\times 100,$$

and the flavonoid concentrations causing 50% *M. aeruginosa* growth inhibition (EC_50_) were estimated by probit analysis. Based on these data, > EC_50_, = EC_50_, and < EC_50_ were tested in further experiments to investigate the physiological response of *M. aeruginosa* to flavonoids.

### 4.3. Pulse-Amplitude-Modulated Fluorescence Analysis of M. aeruginosa Photosynthesis

The photosynthetic activity of *M. aeruginosa* was analyzed by quantitative assessment of photosystem II (PSII) based on chlorophyll fluorescence using a pulse-amplitude-modulated (PAM) fluorescence monitoring system (Water-PAM, Walz, Germany). Each sample was loaded into a cuvette and dark-acclimated for 15 min before analysis. The maximum PSII photochemical efficiency was evaluated by *F_v_*/*F_m_*, where *F_v_* is the difference between the maximum (*F_m_*) and initial (*F_0_*) fluorescence of the darkness-adapted sample. Photosynthetic activity was estimated by the rapid photosynthesis-irradiance response curve (rapid light curve, RLC) technique. A complete RLC was generated by measuring effective quantum yields over a range of nine incremental actinic light intensities (PAR) (0, 37, 121, 178, 274, 404, 618, 924, 1313, and 1815 μmol photons m^−2^ s^−1^) and calculating the corresponding relative electron transport rates (rETRs). Each actinic light irradiance step was applied for 20 s. Based on the model of Eilers and Peeters [43], the photosynthetic parameters alpha (α, initial slope of the light-limited region) and rETR_max_ (maximum rETR) were calculated using the RLC (see Table S4).

### 4.4. Flow Cytometry Analyses of the Flavonoid Effect on Morphology and Membrane System

The effects of flavonoids on the cell volume, cell membrane integrity, and membrane potential levels were analyzed using a flow cytometer (FCM) (Cytomics FC500 MCL System; Beckman Coulter, Brea, CA, USA) equipped with an argon ion laser emitting at 488 nm. Data were collected and statistically analyzed off-line using CXP software (Beckman Coulter). The FCM operating flow rate was constant at 12 μL/min; at least 5000 events were collected for each sample. Before fluorescent probes were loaded, each cell sample was resuspended in phosphate-buffered saline (PBS, pH 7.2) in triplicate.

The *in vivo* chlorophyll auto-fluorescence was determined by a red fluorescence detector (670 nm) as previously described [44] and used to set gating levels to exclude non-cyanobacterial particles. Cell membrane integrity was analyzed by staining with 10 μM propidium iodide (PI; No.F4170, Sigma-Aldrich, Shanghai, China) and 0.5 μM bis-(1,3-dibutylbarbituric acid) trimethine oxonol (DiBAC_4_(3); No.D8189, Sigma-Aldrich) for 10 min. PI penetrates cells with damaged membranes and stains intracellular nucleic acids, producing a bright red fluorescence (620nm) [45], whereas intracellular staining with DiBAC_4_ (3) (green fluorescence, 525 nm) indicates cell membrane depolarization [46]. Data were expressed as the percentage of stained cells relative to the control group.

### 4.5. Data Analysis

All assays were conducted in triplicate. Data were expressed as the mean ± standard deviation. Differences between groups were analyzed by one-way analysis of variance (ANOVA) followed by Tukey’s multiple comparison test. The tests were performed after analyzing the homoscedasticity of variance; *p* < 0.05 was considered statistically significant. All statistical analyses were performed using SPSS 20.0 software (SPSS Inc., Chicago, IL, USA).

Curve fitting of RLCs was obtained by the downhill simplex method of the Nelder-Mead model [47], and standard deviation of parameters was estimated by an asymptotic method. All fittings were tested by analysis of variance (*p* < 0.001), residues being tested for normality and homogeneity of variance and parameter significance by Student’s t test (*p* < 0.05). Comparisons of RLCs were performed based on the method of Ratkowski [26] for non-linear models.

## 5. Conclusions

The response of *M. aeruginosa* to the flavonoids in this study shows that 5,4'-dihydroxyflavone (DHF), apigenin, and luteolin suppress the growth of the cyanobacteria significantly, and the suppressed effects of flavonoid increased, as the concentrations increased. Luteolin disrupts the photosynthetic system and suppresses cyanobacterial growth, whereas 5,4'-DHF and apigenin affect *M. aeruginosa* cell morphology and membrane integrity and function. The experiments described here demonstrate that substitutions of the hydroxyl group in the flavonoid are likely responsible for the different effects on cyanobacterial physiology, which suggests that investigating the relationship between the chemical structure and function of flavonoids could be beneficial to understanding the interactions between flavonoid and cyanobaeterial species in aquatic environments.

## Supplementary Materials

Supplementary materials can be accessed at: http://www.mdpi.com/2072-6651/7/1/0066/s1.
